# Optimizing plasmonic nanoantennas via coordinated multiple coupling

**DOI:** 10.1038/srep14788

**Published:** 2015-10-01

**Authors:** Linhan Lin, Yuebing Zheng

**Affiliations:** 1 Department of Mechanical Engineering, Materials Science & Engineering Program, and Texas Materials Institute, The University of Texas at Austin, Austin, TX 78712, USA

## Abstract

Plasmonic nanoantennas, which can efficiently convert light from free space into sub-wavelength scale with the local field enhancement, are fundamental building blocks for nanophotonic systems. Predominant design methods, which exploit a single type of near- or far-field coupling in pairs or arrays of plasmonic nanostructures, have limited the tunability of spectral response and the local field enhancement. To overcome this limit, we are developing a general strategy towards exploiting the coordinated effects of multiple coupling. Using Au bowtie nanoantenna arrays with metal-insulator-metal configuration as examples, we numerically demonstrate that coordinated design and implementation of various optical coupling effects leads to both the increased tunability in the spectral response and the significantly enhanced electromagnetic field. Furthermore, we design and analyze a refractive index sensor with an ultra-high figure-of-merit (254), a high signal-to-noise ratio and a wide working range of refractive indices, and a narrow-band near-infrared plasmonic absorber with 100% absorption efficiency, high quality factor of up to 114 and a wide range of tunable wavelength from 800 nm to 1,500 nm. The plasmonic nanoantennas that exploit coordinated multiple coupling will benefit a broad range of applications, including label-free bio-chemical detection, reflective filter, optical trapping, hot-electron generation, and heat-assisted magnetic recording.

Plasmonic nanoantennas continue to attract increased attention due to their capability of confining free-space electromagnetic waves into a sub-wavelength region with high field enhancement^1^,^2^,^3^,^4^,^5^,^6^,^7^,^8^,^9^,^10^,^11^,^12^,^13^, which enables a variety of cutting-edge applications such as surface-enhanced Raman spectroscopy (SERS)^14^,^15^,^16^,^17^,^18^,^19^, single-molecule detection^20^,^21^, high-sensitive photodetection^22^, near-field optical trapping^23^, magnetic recording^24^,^25^, and nanoscale light sources^26^. The field enhancement (FE) of the nanoantennas is one of the most important factors for all these applications. Among various designs, plasmonic nanoantennas consisting of adjacent metallic nanoparticles with nanoscale gaps (in particular, bowtie nanoantennas) have shown extremely strong field confinement and enhancement in the gap regions due to the in-plane near-field coupling across the gaps^1^,^3^,^27^. Structural optimization in terms of gap size^1^,^28^,^29^,^30^, antenna size^2^,^29^, antenna shape^2^,^31^, and formation of arrays^23^,^27^,^32^ has been employed to further enhance the concentrated field and tune the spectral response through the in-plane near-field coupling or the far-field in-plane coupling.

Recently, the out-of-plane near-field coupling between a metallic nanoparticle and a metallic thin film separated by a dielectric spacer, known as the metal-insulator-metal (MIM) structure, has also been studied intensely for the nanoantenna applications^15^,^33^,^34^. Unlike bowtie nanoantennas that require sophisticated e-beam lithography or focused ion beam lithography to control the gaps and the coupling, the MIM structures are compatible with the high-throughput thin film processing with the precise control of the spacer thickness down to the atomic scale. The MIM cavity confines light in the form of surface plasmon polaritons (SPPs) along the surface of metallic film and leads to Fabry-Perot resonances along the direction perpendicular to the film^34^,^35^,^36^,^37^,^38^,^39^,^40^,^41^. Besides the FE^40^, other intriguing optical responses were observed in the MIM nanosystems for enhanced applications, including dual resonances for SERS^15^. So far, most of the designs on plasmonic nanoantennas have been limited to the exploitation of a single type of the three coupling effects, i.e., the in-plane near-field coupling (as in single bowtie structures), the in-plane far-field coupling (as in nanoparticle arrays), and the out-of-plane near-field coupling (as in MIM structures). Considering the unique optical characteristics and the feasibility for an independent tuning of each type of coupling, we propose to exploit the coordinated multiple coupling in plasmonic nanoantennas to optimize the spectral tunability and the local field enhancement.

To demonstrate this concept, we apply finite-difference time-domain (FDTD) simulations to fully study the coupling behavior and optical properties in the bowtie nanoantenna arrays (BNAs) with MIM configuration. Through designing and tuning the multiple coupling, i.e., the in-plane near-field coupling, the in-plane far-field coupling, and the out-of-plane coupling, in the proposed arrays, we achieve the further enhancement of electric field and the large tunability of spectral responses. To further demonstrate the practical applications of the coordinated multiple coupling in the plasmonic nanoantenna arrays, we design a refractive index sensor with an ultra-high figure-of-merit and a narrow-band near-infrared plasmonic absorber with high absorption efficiency based on the optimized BNAs with MIM configuration. Applications of the ultra-intense “hot spots” in the BNAs to near-field optical trapping, heat-assisted magnetic recording and hot-electron generation are also discussed.

## Results

**Figure 1:**
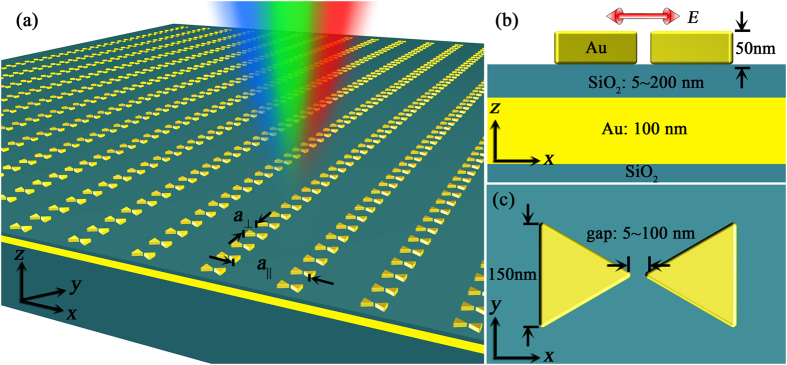
Schematic of the plasmonic BNAs and single bowtie antenna with MIM configuration. (**a**) Schematic of the Au BNAs with MIM configuration. (**b**) Front view and (**c**) top view of a single bowtie nanoantenna. The thickness and length of the Au equilateral triangles are 50 nm and 150 nm, respectively. The gap between the tips of the two triangles ranges from 5 to 100 nm. A SiO_2_ spacer layer with the thickness ranging from 5 to 200 nm lies below the BNAs. An Au thin film is sandwiched between the spacer layer and the SiO_2_ substrate. The thickness of the Au thin film is set as 100 nm, which totally blocks the light transmission through the structure.

The BNAs with MIM configuration are schematically displayed in Fig. 1a, with the front view and top view shown in Fig. 1b,c, respectively. The side length of the equilateral triangle of Au is 150 nm. The gap size of the bowtie structure ranges from 5 to 100 nm, which tunes the in-plane near-field coupling. The SiO_2_ spacers with different thicknesses *t* = 5, 10, 20, 50, 100 and 200 nm are used to demonstrate the different out-of-plane coupling behavior. The optically thick Au film (100 nm) is embedded between the spacer and the SiO_2_ substrate. *a*_||_ and *a*_⊥_ indicate the lattice constants that are parallel and vertical to the polarization direction of the incident light, respectively, which control the far-field in-plane coupling known as plasmonic-photonic coupling in the periodic arrays.

### Single bowtie nanoantennas with MIM configuration

**Figure 2:**
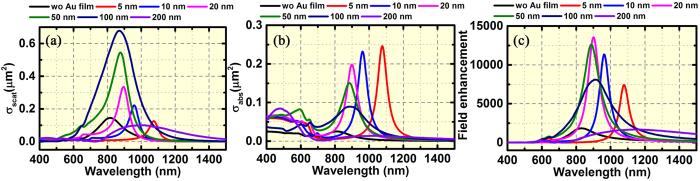
Spectral responses of the single bowtie nanoantenna with and without MIM configuration. (**a**) Scattering cross section, (**b**) absorption cross section, and (**c**) FE factor (|*E*|^2^/|*E*_0_|^2^) of a single bowtie nanoantenna with MIM configuration as a function of the spacer thickness ranging from 5 to 200 nm. The gap size of the bowtie nanoantennas is 10 nm. The results for the bowtie nanoantenna without MIM configuration are included for comparisons. The electric field intensity in (**c**) was measured in the middle of the bowtie gap.

**Figure 3:**
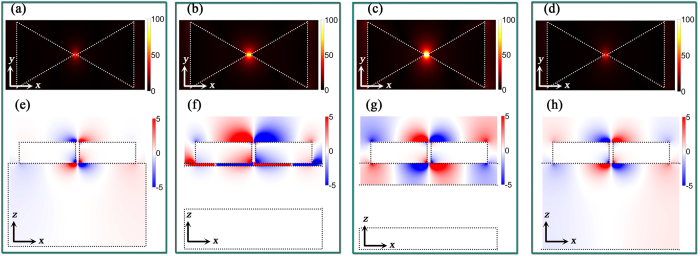
Electric field distributions in the single bowtie antenna with and without MIM configuration. (**a**–**d**) show the |*E*_*x*_| distributions in *xy* plane, which is 25 nm above the SiO_2_ spacer and (**e**–**h**) show the *E*_*z*_ distributions in *xz* plane through the center of the tip-to-tip bowtie regions. (**a**,**e**) bowtie nanoantennas without MIM configuration (λ = 817 nm). (**b**,**f**) bowtie nanoantennas with MIM configuration (*t* = 5 nm and λ = 1,074 nm). (**c**,**g**) bowtie nanoantennas with MIM configuration (*t* = 50 nm and λ=877 nm). (**d**,**h**) bowtie nanoantennas with MIM configuration (*t* = 200 nm and λ = 1,000 nm). The gap size of the bowtie nanoantennas is 10 nm.

Before incorporating the far-field in-plane coupling in the BNAs with MIM configuration, we study both the in-plane and out-of-plane coupling in the single bowtie nanoantenna with MIM configuration. We compare the scattering and absorption cross section of the bowtie nanoantennas with and without MIM configuration. As shown in Fig. 2a,b, a strong out-of-plane coupling is obtained in the MIM cavity, leading to an obvious redshift of the resonance wavelength at a spacer thickness of 5 nm. The peak wavelength makes a continuous blueshift with an increased scattering cross section and a decreased absorption cross section when the spacer thickness is increased from 5 to 100 nm. Both the absorption and scattering cross sections drop dramatically when *t* reaches 200 nm. The absorption cross section is almost becoming zero and radiative scattering dominates the extinction spectrum within the wavelength range from 800 nm to 1,500 nm. The dependence of the light scattering and absorption in the bowtie antenna is further clarified by the Poynting vector distributions (Supplementary Fig. S1), revealing the confinement of light in the spacer when *t* = 5 nm (corresponding to the high absorption cross section) and a significant scattering for larger spacer thickness (*t* = 50 and 200 nm). It was argued that the gap surface plasmon (GSP) dominates the out-of-plane coupling when the thickness of the spacer is small, while the SPP is dominant when the thickness is larger^35^,^36^,^39^,^41^. Our results support this argument. When *t* = 5 nm, the multiple reflection in the spacer leads to a strong near-field coupling between the bowtie nanoantenna and the Au film and results in the GSP in the spacer, where the electromagnetic field is confined (see Fig. 3b,f). This out-of plane coupling can be attributed to the near-field interaction between the bowtie nanoantenna and its image in the Au film^42^, which is similar to the GSP in the neighboring metallic nanoparticles. Since the “hot spots” are confined in the spacer, radiative loss is lower than that of the bowtie nanoantenna without the MIM configuration (Fig. 2a). The increase of the spacer thickness decreases the near-field coupling strength in the spacer (Fig. 3), causing blueshift in the resonance wavelength and broadening in the spectral linewith.

We further study the electric field intensity in the middle of the bowtie gap as a function of the spacer thickness (Fig. 2c). It should be noted that the field in this gap region arises from the in-plane near-field coupling between the two tip-to-tip Au triangles and the out-of-plane component is almost zero in the gap (see *E*_z_ distributions in Fig. 3). However, this in-plane coupling is strongly dependent on the out-of-plane coupling induced by the MIM configuration. As shown in Fig. 2, there is no correspondence between the spacer thickness for the maximum FE and that for the maximum scattering and absorption. The highest intensity of electric field is obtained when *t* = 20 nm, which is 6 times higher than that of bowtie nanoantenna without the MIM configuration. This field enhancement arises from the interactions between the bowtie nanoantenna and the reflected field from the Au film, i.e., the radiation field from the image in the Au film^40^. Different from the GSP in the spacer, in which the field intensity in the spacer is reduced when the spacer thickness is increased. The radiation field from the image of the bowtie antenna in the Au film interacts with the radiation field from the real bowtie antenna and modify the radiation quality factor (Q factor), when the radiation Q factor equals to the absorption Q factors, the maximum FE factor can be obtained (also see Supplementary Fig. S2).

To further examine the coupling behavior in the MIM configuration, we study the distributions for the in-plane “hot spots” (|*E*_*x*_|) and the out-of-plane component (*E*_*z*_) in the single bowtie nanoantenna. It should be noted that the former is determined by gap size (in-plane coupling) and the phase difference between the bowtie nanoantennas and its image in the Au film, while the latter originates from the cavity formed in the spacer. However, both of them are tunable by controlling the spacer thickness. As shown in Fig. 3a,e, when the bowtie nanoantenna was directly placed on the SiO_2_ substrate (*t* = ∞), the in-plane near-field coupling generates the “hot spots” in the gap (Fig. 3a), which also leads to the out-of-plane “hot spots” above and below the tips of the triangles due to charge distributions. When *t* = 5 nm, besides the enhanced in-plane “hot spots” in the bowtie gap (Fig. 3b), a Fabry-Perot cavity arises along *z* axis (referring to Fig. 1) in the spacer and an out-of-plane GSP with high field intensity is observed (Fig. 3f). The enhanced out-of-plane field components are observed below both the tips and the ridges of the triangles. Interestingly, another pair of out-of-plane “hot spots” is observed on the top of the ridges when the MIM configuration is introduced. The larger spacer turns MIM into insulator-metal-insulator (IMI) configuration where SPP (rather than GSP) dominates the coupling in the spacer and leads to rapid attenuation of the field intensity in the spacer (Fig. 3g). The in-plane propagating SPP can be observed in Fig. 3g, while the intensity decreases when *t* reaches 200 nm (Fig. 3h). The evolution of the in-plane “hot spots” intensity (Fig. 3a–d) matches well with the field intensity shown in Fig. 2c.

### Coupling behavior in the BNAs with MIM configuration

The plasmonic-photonic coupling in the metallic nanoparticle arrays has attracted strong interests^43^,^44^,^45^,^46^,^47^. The far-field radiative coupling between each unit in the arrays leads to collective resonance, enhancing the electric field intensity, suppressing the radiative damping, and narrowing the linewidth of spectra. This collective behavior is known as lattice plasmon resonances (LPRs). Both orthogonal^43^,^44^,^45^,^46^ and parallel coupling^48^,^49^,^50^,^51^ have been studied recently. The former is achieved by coupling the in-plane “hot spots” (e.g., the dipole mode in the Au nanodisc or nanorod arrays^45^,^46^) with the diffraction waves while the latter arises from the coupling between the out-of-plane “hot spots” (e.g., the out-of-plane components induced by the in-plane dipole modes or higher order modes^50^,^51^) and the diffraction waves. Recently, we have shown that the manipulation of “hot spots” location for the tuning of the radiative scattering from the localized modes and the maintenance of robust diffraction waves in the arrays paves the way towards the versatile engineering of the plasmonic-photonic coupling^51^. In the next two sections, we demonstrate that the engineering of both out-of-plane and in-plane near-field couplings tunes the far-field coupling, maximizing the electric field intensity in the bowtie gap regions and increasing the spectral response of the BNAs with MIM configuration.

**Figure 4:**
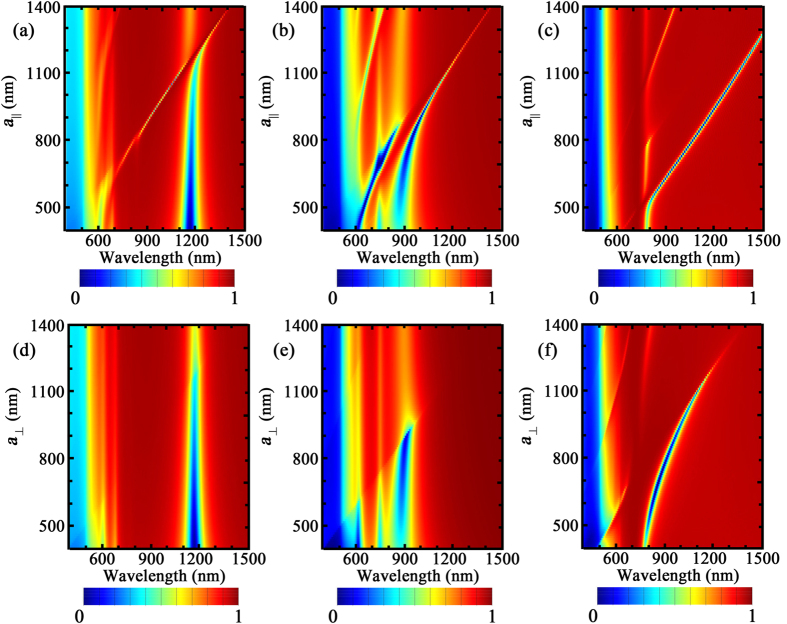
Spectral responses of the BNAs with MIM configuration. Simulated reflection spectra of the BNAs with MIM configuration as a function of (**a**–**c**) *a*_||_ and (**d**–**f**) *a*_⊥_ for different spacer thicknesses: (**a**,**d**) *t* = 5 nm, (**b**,**e**) *t* = 50 nm, and (**c**,**f**) *t* = 200 nm. The gap size of the bowtie nanoantennas is 10 nm.

**Figure 5:**
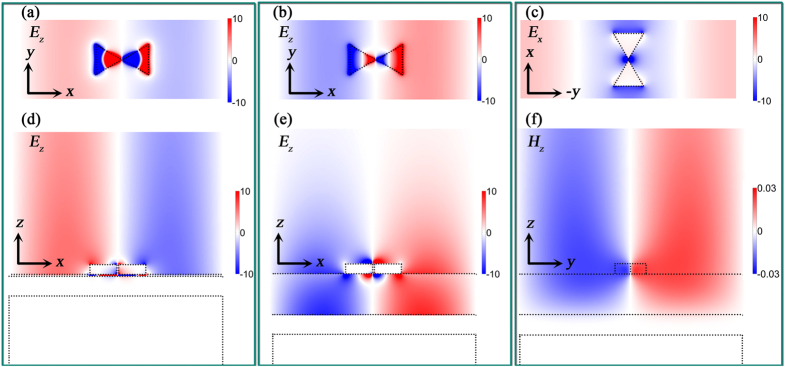
Electromagnetic field distributions in the BNAs with MIM configuration. Distributions of (**a**,**b**) the real parts of *E*_*z*_ in the *xy* plane, which is 3 nm below the bottom of the BNAs, (**c**) the real parts of *E*_*x*_ in the *xy* plane, which is 25 nm above the spacer, (**d**,**e**) the real parts of *E*_*z*_ in the *xz* plane and (**f**) the real parts of *H*_*z*_ in the *yz* plane through the center of the tip-to-tip regions for the BNAs with MIM configuration: (**a**,**d**) *t* = 5 nm, *a*_⊥_ = 400 nm, *a*_||_ = 1040 nm, λ = 1045 nm; (**b**,**e**) *t* = 200 nm, *a*_⊥_ = 400 nm, *a*_||_ = 1000 nm, λ = 1255 nm; (**c**,**f**) *t* = 200 nm, *a*_||_ = 400 nm, *a*_⊥_ = 1,100 nm, λ = 1,109 nm. The gap size of the bowtie nanoantennas is 10 nm.

The parallel plasmonic-photonic coupling behavior in the BNAs with MIM configuration is displayed in Fig. 4a–c where *a*_⊥_ = 400 nm, *a*_||_ ranges from 400 to 1,500 nm, and *t* = 5, 50 and 200 nm, respectively. The parallel coupling in the BNAs without MIM configuration is very weak (Supplementary Fig. S3b). However, engineering of the out-of-plane coupling with the MIM configuration leads to robust parallel coupling. As shown in Fig. 4a, the coupling occurs at the higher energy side of the localized surface plasmon mode when *t* = 5 nm. Although the absorption cross section dominates the extinction spectrum with the weak radiative scattering in the single bowtie structure (Fig. 2a,b), the out-of-plane coupling enhances the “hot spots” on the top of the ridges (Fig. 3f), which enables the parallel coupling with the horizontal propagating *E*_z_ components 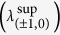 in the diffraction waves (Fig. 5d). However, due to strong near-field coupling in the spacer and the high absorption efficiency, the “hot spots” in the spacer cannot couple with the diffraction waves (Fig. 5a). Increasing the lattice constant *a*_||_ redshifts the 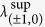 to the longer wavelength. When 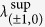 is located at the longer wavelength side of the localized mode, its coupling with the out-of-plane “hot spots” above the tips of the triangles is achieved. However, the small scattering cross section at spacer thickness *t* = 5 nm leads to the low parallel plasmonic-photonic coupling efficiency and lower extinction efficiency is observed above 1,200 nm.

We find that an increase of the spacer thickness improves the parallel coupling efficiency. The increase of spacer thickness enhances the weight of radiative scattering of individual bowtie antenna (Fig. 2a), which is helpful to improve the in-plane multiple scattering. Fig. 4b shows a more robust coupling between the localized mode with the diffraction waves when the diffraction orders are located at the lower energy side of the localized mode. Moreover, the increased thickness reduces the GSP in the spacer and the SPP becomes dominant. Specifically, the periodic arrays make it possible to couple the SPP in the spacer with the diffraction waves and further enhance the in-plane radiative interactions. Also, the broadening bandwidth in the scattering spectrum (Fig. 2a) with spacer thickness enables the robust coupling in a wide range of working wavelength. An extremely robust coupling is observed when *t* = 200 nm (Fig. 4c) where high coupling efficiency is obtained when *a*_||_ is increased from 600 to 1,300 nm. The field distributions in Fig. 5b,e show that both the “hot spots” below the ridges and above the tips of the triangles can couple with the diffraction waves. It is considered that the spacer with 200 nm thickness is insufficient to support the diffraction waves for the far-field coupling^50^. However, the coupling between the SPP in the spacer and the diffraction waves generates the strong in-plane propagating electric field, which strengthens the radiative scattering in the spacer and enables robust far-field coupling. The intense *E*_*z*_ components in the spacer and the decay of *E*_*z*_ away from the surface of the Au film confirm the coupling between the SPP and the diffraction waves (Fig. 5e). In addition, the wavelength of the reflection dips is consistent with *n*_sub_*a*_||_ (Fig. 4c), indicating that the in-plane scattering is dominant in the spacer.

The orthogonal coupling behavior in the BNAs with MIM configuration is displayed in Fig. 4d–f where *a*_||_ = 400 nm, *a*_⊥_ ranges from 400 to 1,500 nm, and *t* = 5, 50 and 200 nm, respectively. Different from the parallel coupling, the in-plane “hot spots” (in-plane GSP in the bowtie gap) with high scattering efficiency are required to achieve robust orthogonal coupling. No plasmonic-photonic coupling is observed in the BNAs with small spacer thickness, and an increase of the spacer thickness enables the coupling between the GSP and 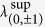. In contrast to the BNAs without MIM configuration (Supplementary Fig. S3a), the orthogonal coupling with the substrate diffraction orders cannot be achieved in the BNAs with MIM configuration because the thick Au film cuts off the substrate diffraction orders and the thin spacer cannot support the new diffraction orders. When *t* = 5 nm, the scattering cross section of the in-plane “hot spots” (in-plane GSP) is extremely low and the extinction is dominated by the absorption cross section (Fig. 2a,b) due to the strong near-field coupling in the spacer (also see the Poynting vector in Supplementary Fig. S1a), suppressing the plasmonic-photonic coupling. An increase of the spacer thickness enhances the radiative scattering and reduces the absorption in the structures, leading to the orthogonal coupling. Figure 4b reveals that a weak coupling between the GSP and 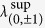 occurs. When *t* = 200 nm, radiative scattering dominates, enabling the robust coupling between the GSP and 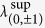. Figure 5c,f reveal the coupling between the in-plane “hot spots” in the gap region and the in-plane propagating *H*_*z*_.

### Maximum field enhancement

The FE in the gap regions of the bowtie nanoantennas is one of the most important factors for their applications. Much work has been carried out to improve the FE factor in the gap^40^,^52^. In this section, we demonstrate that the plasmonic-photonic coupling in the BNAs with MIM configuration can be harnessed to maximize the FE in the gap. With the maximum FE, the BNAs have potential applications in near-field optical trapping^23^,^53^, heat-assisted magnetic recording^25^, hot-electron generation^54^,^55^, and strong-field photoemission^56^.

**Figure 6:**
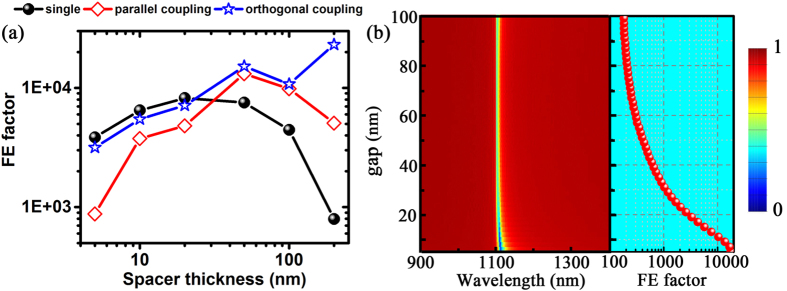
FE factors of the single bowtie antenna and BNAs with MIM configuration. (**a**) FE factor of the single bowtie antenna and BNAs with MIM configuration as a function of spacer thickness. *a*_⊥_ = 400 nm, *a*_||_ = 1,045, 900, 800, 900, 1,000 and 1,000 nm are chosen for t = 5, 10, 20, 50, 100 and 200 nm, respectively, for parallel coupling. *a*_||_ = 400 nm, *a*_⊥_ = 1,100, 1,100, 1,100, 900, 900 and 1,100 nm are chosen for t = 5, 10, 20, 50, 100 and 200 nm, respectively, for orthogonal coupling. The gap size of the bowtie nanoantennas is 10 nm. The field intensity is measured 3 nm above the tips of the nanoantennas. (**b**) Simulated reflection spectra and FE factors of the BNAs with MIM configuration as a function of the gap size. The parameters of the BNAs with MIM configuration: *t* = 200 nm, *a*_||_ = 400 nm, *a*_⊥_ = 1,100 nm. The field intensity is measured in the middle of the bowtie gap that is 3 nm above the top-surface level of the nanoantenna.

Figure 6a summarizes the FE factors for single bowtie nanoantenna, BNAs with parallel coupling and BNAs with orthogonal coupling at different spacer thicknesses. The resonance peak wavelengths with the highest extinction efficiency were used for the simulations. The detectors are located 3 nm above the tips of the nanoantennas to measure the FE factor. At small spacer thicknesses, the parallel coupling between the “hot spots” above the ridges of the BNAs and 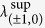 leads to a significant field enhancement along the ridges. The FE factor at the tip regions of the arrays is lower than that of the single bowtie nanoantennas with MIM configuration (Fig. 5d). The larger spacer thickness (*t* = 50 nm) increases the coupling between the out-of-plane “hot spots” above the triangle tips and 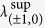 (Fig. 4b). The further enhancement of the electric field above the tips leads to a high FE factor of 13,000. Although a robust coupling is obtained between the “hot spots” above the tips and 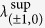 for a spacer thickness of 200 nm (Fig. 4c), the lower FE factor in the individual bowtie nanoantennas (~900) limits the final enhancement. Still, an FE factor of 6,000 is much higher than that in BNAs without MIM configuration (Supplementary Fig. S4).

Another strategy to maximize the FE factor is to strengthen the in-plane resonance, i.e., the in-plane GSP in the gap by introducing the orthogonal plasmonic-photonic coupling. As shown in Fig. 6, the strong orthogonal coupling at the larger spacer thickness enhances the maximum FE factor from 3,000 (*t* = 5 nm) to 22,000 (*t* = 200 nm). This is 22 times higher than that in the single bowtie nanoantenna without MIM configuration and 8 times higher than that in the BNAs without MIM configuration (Supplementary Fig. S4). It is also noted that introducing the orthogonal coupling in the BNAs without MIM configuration shows a 3 times enhancement compared with that in the single bowtie antenna. However, a 6 times enhancement in the parallel coupling and 24 times enhancement in the orthogonal coupling is achieved compared with the FE factor in the single structure when MIM configuration is utilized. Therefore, the MIM configuration can improve the far-field coupling efficiency in the BNAs by inducing the effective coupling between the SPP and the diffraction waves and strength the in-plane scattering.

The size variation of the gap between the tip-to-tip triangles can modify the extinction efficiency and the FE factor of the BNAs where the far-field coupling occurs. We study the reflection spectra of the BNAs with MIM configuration as a function of the gap size where the robust orthogonal coupling exists. As shown in Fig. 6b, the extinction efficiency increases from 58% to 81% when the gap size decreases from 100 nm (without in-plane near-field coupling) to 15 nm. Meanwhile, the FE factor is improved up to 2 orders of magnitude. Furthermore, the extinction efficiency changes from 65% to 100% as a function of bowtie gap size in the BNAs with MIM configuration at the parallel plasmonic-photonic coupling (Supplementary Fig. S5).

### High-performance refractive index sensors

Metallic nanoparticle arrays with the plasmonic-photonic coupling have been explored as high-performance refractive index sensors due to the high sensitivity to the surrounding refractive index and the narrow linewidth of the LPRs leading to high figure-of-merit (FOM)^57^,^58^,^59^,^60^. However, the LPR-based refractive index sensors reported so far have limited dynamic range and FOM (20 ~ 30)^59^,^60^. Recently, a high FOM of 108 has been achieved in the Au mushroom arrays, however, with the low signal-to-noise ratio (SNR)^58^. In this section, we design and analyze high-performance refractive index sensors based on the BNAs with MIM configuration, which features high FOM, high SNR and a wide range of detectable analyte refractive index.

**Figure 7:**
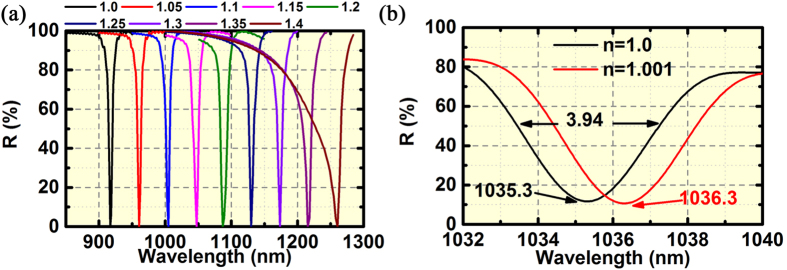
Refractive-index sensing of the BNAs with MIM configuration based on parallel coupling. (**a**) Normalized reflection dips of the BNAs (*t* = 5 nm, *a*_⊥_ = 400 nm and *a*_||_ = 900 nm, the gap size of the bowtie nanoantennas is 10 nm) as a function of the superstrate refractive index ranging from 1.0 to 1.4. (**b**) Zoom-in reflection spectra of the BNAs (*t* = 5 nm, *a*_⊥_ = 400 nm and *a*_||_ = 1,030 nm, the gap size of the bowtie nanoantennas is 10 nm) when superstrate refractive index changes from 1.0 to 1.001.

As shown in Fig. 4a, a robust coupling between the out-of-plane “hot spots” above the ridges of the BNAs and 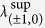 leads to an ultra-narrow linewidth in the reflection spectra. Therefore, we develop the refractive index sensors based on the parallel plasmonic-photonic coupling in the BNAs with MIM configuration where the spacer thickness *t* = 5nm. Figure 7a shows the normalized reflection spectra as a function of the superstrate refractive index. Using the full-width half-maximum (FWHM) at *n* = 1.0, we obtain an FOM of 148. The plasmonic-photonic far-field coupling is robust when the refractive index changes from 1.0 to 1.3. At *n* = 1.35, the coupling between 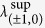 and GSP is observed, resulting in a Fano-like spectrum with a wider linewidth.

We further optimize the structure of BNAs to achieve the stronger plasmonic-photonic coupling, leading to the higher extinction efficiency and narrower linewidth. Using the optimized parameters of *a*_⊥_ = 400 nm and *a*_||_ = 1,030 nm, we obtain the FWHM of 3.94 nm and the wavelength sensitivity of 1000 nm per refractive index unit (Fig. 7b), leading to a higher FOM of 254. We also study the sensors based on the orthogonal coupling (Supplementary Fig. S6), which exhibit a high FOM of 108 with limited dynamic range. So far, the refractive index sensors based on the parallel coupling in the BNAs with MIM configuration have shown the highest FOM, a high SNR and a large dynamic range.

### Perfect narrow-band absorbers

Nanostructured surfaces with high narrow-band absorption efficiency find various applications in optical filters, monochromatic photodetectors, thermal emitters, and optical modulators^61^,^62^,^63^,^64^,^65^. With the capability of surface plasmons in concentrating light at the nanoscale and converting it into thermal energy, metallic nanoparticles have advantages for absorber applications. However, it has remained challenging to develop plasmonic absorbers with narrow band, high efficiency and a wide range of working wavelength. In this section, we design the BNAs with MIM configuration to work as a perfect narrow-band absorber with the working wavelength tunable from 900 nm to 1,500 nm.

**Figure 8:**
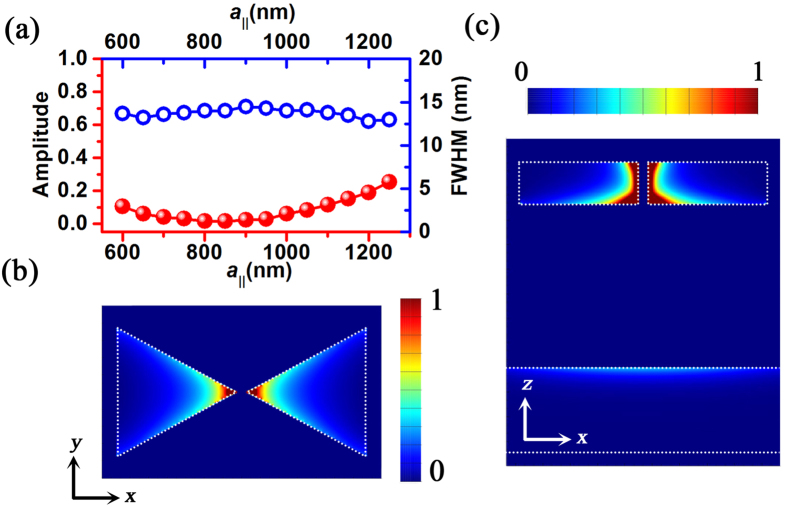
Narrow band absorbers based on the BNAs with MIM configuration. (**a**) Amplitude and FWHM of the reflection spectra of the BNAs with MIM configuration as a function of the lattice constants with *t* = 200 nm, *a*_⊥_ = 400 nm and *a*_||_ ranging from 600 to 1,250 nm. Normalized absorption power distributions in the BNAs with MIM configuration (*t* = 200 nm, *a*_⊥_ = 400 nm and *a*_||_ = 1,000 nm at λ = 1,255 nm) in both the (**b**) *xy* plane and (**c**) *xz* plane through the center of the bowtie nanoantennas. The maximum color scale in (**c**) is 2 times higher than that in (**b**). The gap size of the bowtie nanoantennas is 10 nm.

Figure 8a shows the amplitude and FWHM of the reflection spectra of the BNAs with MIM configuration (*t* = 200 nm) as a function of the lattice constants *a*_||_. The robust parallel plasmonic-photonic coupling leads to a promising narrow-band absorber. When *a*_||_ is increased from 600 nm to 1,250 nm, the FWHM is maintained at ~13 nm. The absorption efficiency reaches 100% when *a*_||_ ranges from 750 nm to 950 nm. The Q factor is 67 at the shorter wavelength (λ = 870 nm and *a*_||_ = 600 nm) and 114 at the longer wavelength (λ = 1,480 nm and *a*_||_ = 1,250 nm). Figure 8b,c displays the two-dimensional distribution of the light absorption. The highest power intensity absorbed is located at the tips of the bowtie nanoantennas with the highest field intensity (also see Supplementary Fig. S8). This further confirms that the gap size is crucial to achieve high absorption efficiency, which is consistent with the results in Fig. 6b and Supplementary Fig. S5. The narrow-band absorption characteristics of the BNAs with other spacer thickness in parallel coupling and orthogonal coupling are summarized in Supplementary Fig. S7. The robust orthogonal coupling in the BNAs with *t* = 200 nm also leads to a high-performance narrow-band absorber (Supplementary Fig. S7c). However, compared with the BNAs with parallel coupling, the BNAs with orthogonal coupling have the larger FWHM and thus the lower Q factor.

## Discussion

With the capability of efficiently converting light from free space into sub-wavelength scale and vice versa, plasmonic nanoantennas find a wide range of applications in nanophotonics. Exploiting multiple coupling in plasmonic nanoantenna arrays paves the way towards both enhancement and versatile tuning of their characteristics in order to meet the requirements of different applications. Taking BNAs with MIM configuration as an example, we have demonstrated the feasibility and effective strategy for the synergistic integration of multi-dimensional near- and far-field coupling to achieve both the large tunability of spectral responses and the maximization of FE factor. Moreover, we have designed and analyzed both high-performance refractive index sensors and perfect narrow-band absorbers by exploiting multiple coupling in the BNAs with MIM configuration. We believe that our concept of multiple coupling is applicable to general plasmonic nanosystems for their enhanced applications in near-field optical trapping, heat-assisted magnetic recording, plasmonic filter, strong-field photoemission, and hot-electron generation.

## Methods

The simulations were performed using a commercial software (FDTD solutions, Lumerical Solutions). The total-field scattered-field (TFSF) source was employed in the simulations of single bowtie antenna. The plane wave was launched normally from the top of the bowtie antenna. Two detector boxes were placed inside and outside the TFSF source to measure the absorption and scattering cross sections, respectively. Perfect matching layers (PML) were applied in all the boundaries. Symmetric and asymmetric boundary conditions were applied in the simulations of BNAs with or without MIM configuration. The linear-polarized plane wave light source was launched from the top side of the BNAs. Two detectors were placed above and below the BNAs to obtain the reflection and transmission spectra, respectively. An ultra-fine mesh size (1 nm) was used in the simulations. The refractive indices of *n*_sub_ = 1.45 and *n*_sup_ = 1.0 are chosen for SiO_2_ and air to generate an asymmetric environment. The optical constants of Au were taken from Johnson and Christy^66^.

The refractive index sensing was simulated by changing the refractive index of the environment and measuring the reflection spectra. The absorbed power was calculated by 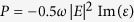, in which *ω* = 2*πf*, *f* is the frequency of the light, |*E|*^2^ is the electric field intensity, Im(ε) is imaginary part of the dielectric function.

## Additional Information

**How to cite this article**: Lin, L. and Zheng, Y. Optimizing plasmonic nanoantennas via coordinated multiple coupling. *Sci. Rep*. **5**, 14788; doi: 10.1038/srep14788 (2015).

## Supplementary Material

Supplementary Information
