# Supplementary material for: Optimizing plasmonic nanoantennas via coordinated multiple coupling
Source: Sci Rep. 2015 Oct 1;5:14788. doi: 10.1038/srep14788 (PMC4589761; doi:10.1038/srep14788)
Supplement: Supplementary Information [file srep14788-s1.doc]

**Supplementary Information**

**Optimizing plasmonic nanoantennas via coordinated multiple coupling**

Linhan Lin & Yuebing Zheng

Department of Mechanical Engineering, Materials Science & Engineering Program, and Texas Materials Institute, The University of Texas at Austin, Austin, TX 78712, USA.

Correspondence and requests for materials should be addressed to Y.Z. (email: [zheng@austin.utexas.edu](mailto:zheng@utexas.edu)).

Supplementary Fig. S1 shows the Poynting vector distributions for different single bowtie nanoantennas with MIM configuration where spacer thickness *t*=5, 50 and 200 nm, respectively. When *t*=5 nm, the light is mainly confined in the SiO_2_ waveguide due to the strong near-field out-of-plane coupling and the gap surface plasmon (GSP) in the spacer. The increase of *t* leads to the significant scattering due to the weaker field localization in the spacer, as shown in Supplementary Figs. S1b and c.


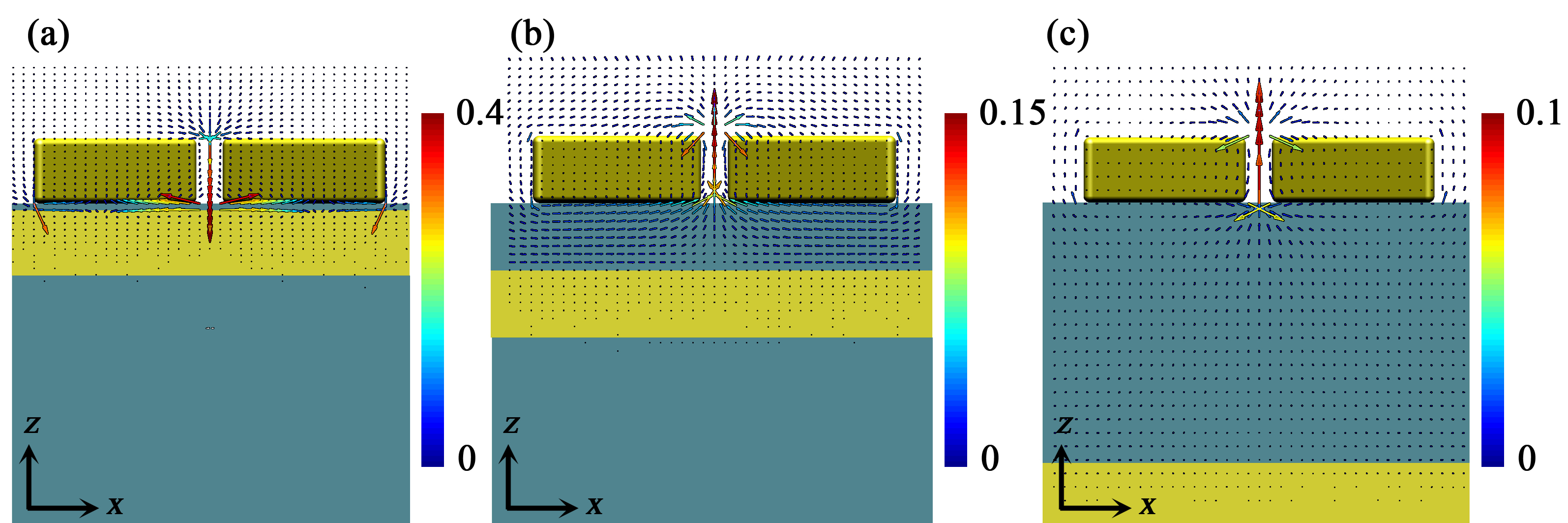


**Supplementary Figure S1 Poynting vector distributions of different single bowtie nanoantennas with MIM configuration.** The spacer thickness *t* is tuned: (**a**) *t*=5 nm, (**b**) *t*=50 nm, (**c**) *t*=200 nm. The gap size of the bowtie nanoantennas is 10 nm.

Due to the radiative interactions between the bowtie nanoantenna and its image in the Au film, the constructive and destructive interferences alternate when the spacer thickness is increased. An evolution from constructive to destructive interference is observed when the spacer thickness is changed from 100 to 200 nm, as shown in Supplementary Fig. S2. The increase of the spacer thickness to 400 nm leads to the high extinction cross section and a high FE factor due to the Q factor matching between the scattering and absorption spectra.


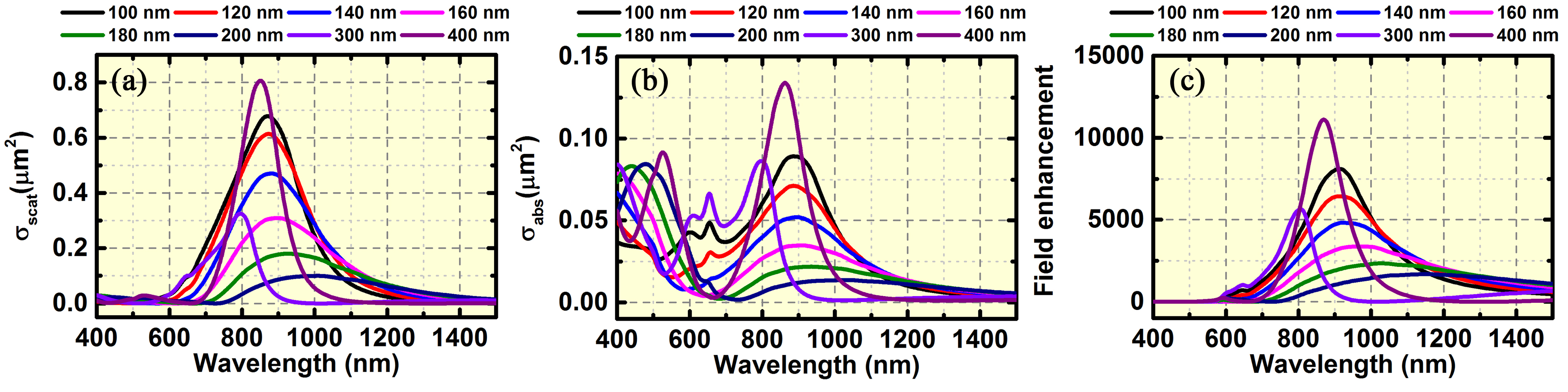


**Supplementary Figure S2 Spectral responses of the single bowtie nanoantennas with and without MIM configuration.** (**a**) Scattering cross section, (**b**) absorption cross section, and (**c**) FE factor (|*E*|2/|*E*_0_|^2^) of a single bowtie nanoantenna with MIM configuration as a function of the spacer thickness ranging from 100 to 400 nm. The gap size of the bowtie nanoantennas is 10 nm. The results for the bowtie nanoantennas without MIM configuration are included for comparisons. The electric field intensity in (c) was measured in the middle of the bowtie gap.

The orthogonal and parallel plasmonic-photonic coupling behaviors in the BNAs without MIM configuration is shown in Supplementary Fig. S3. In Supplementary Fig. S3a, the coupling between the in-plane GSP and the (0, ±1) substrate diffraction orders is obtained when a_⊥_=600 nm. In contrast, only the weak coupling between the in-plane GSP and the (±1, 0) superstrate diffraction orders is observed in Supplementary Fig. S3b.


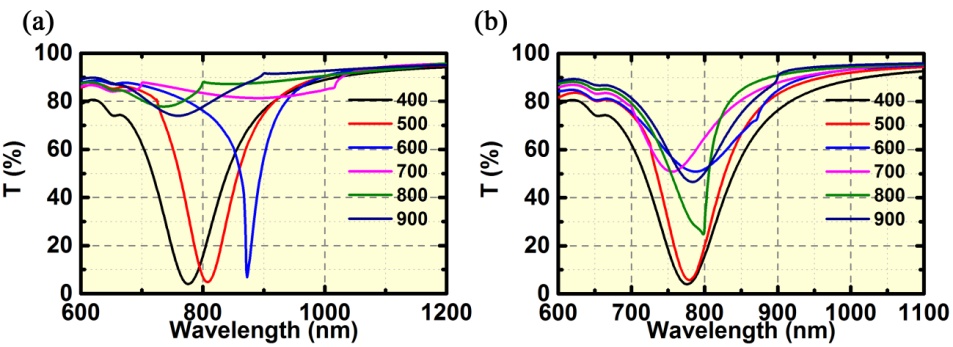


**Supplementary Figure S3 Plasmonic-photonic coupling in BNAs without MIM configuration.** The orthogonal and parallel plasmonic-photonic coupling in the two series of BNAs without MIM configuration where (**a**) *a*_||_=400 nm, *a*_⊥_ ranging from 400 to 1,500 nm, and (**b**) *a*_⊥_=400 nm, a_||_ ranging from 400 to 1,500 nm. The gap size of the bowtie nanoantennas is 10 nm.

Supplementary Fig. S4 shows the field enhancement distributions in the *xy* planes (see Fig. 1) above and below the bowtie nanoantennas with and without the plasmonic-photonic coupling. Two detectors were placed 3 nm above and below the nanoantennas to measure the electric field, as shown in Fig. S4a. The electric field intensity (|*E*|2/|*E*_0_|^2^) of a single bowtie nanoantenna is displayed in Supplementary Fig. S4b, where the maximum FE factors of 1,000 and 3,500 exist at the top and bottom. Upon the orthogonal coupling, a significant FE improvement is observed, with maximum FE factors of 3,200 and 8,400 at the top and bottom, respectively.


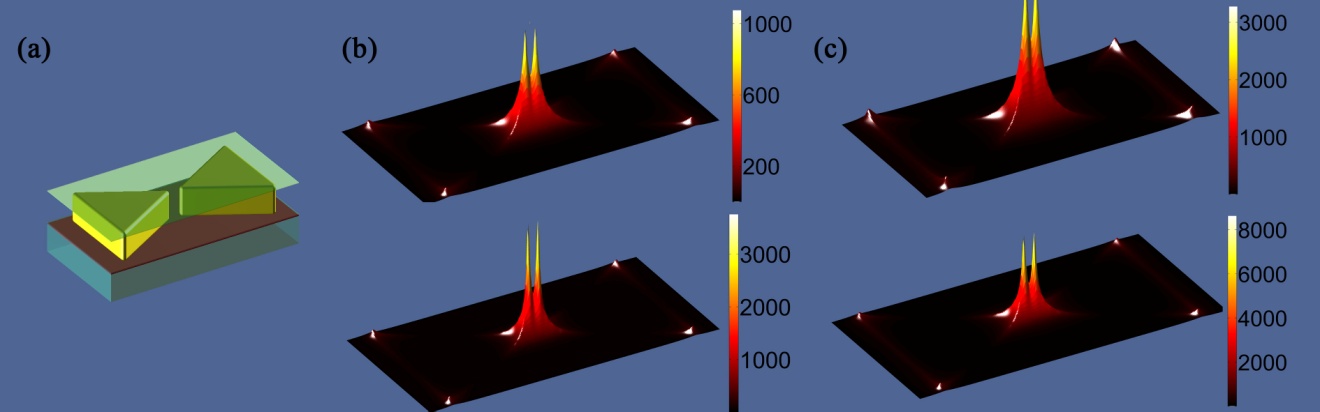


**Supplementary Figure S4 Electric field mapping for single bowtie antenna and BNAs without MIM configuration.** (**a**) Schematic of a single bowtie nanoantenna with two detectors placed in the *xy* plane 3 nm above (green color) and below (red color) the nanoantenna to measure the electric field. The |*E*|2/|*E*_0_|^2^ distributions at the two detection planes in (**a**) for (**b**) a single bowtie nanoantenna at *λ*=840 nm and (**c**) BNAs with *a*_||_ =400 nm, *a*_⊥_=600 nm at* λ*=873 nm. The gap size of the bowtie nanoantennas is 10 nm.

Supplementary Fig. S5 shows the reflection spectra of BNAs with MIM configuration as a function of the bowtie gap size where the robust parallel coupling occurs. The extinction efficiency increases from 65% to 100% with a dramatic enhancement of the FE factor when the gap size is reduced from 100 nm (without the in-plane near-field coupling) to 10 nm and below.


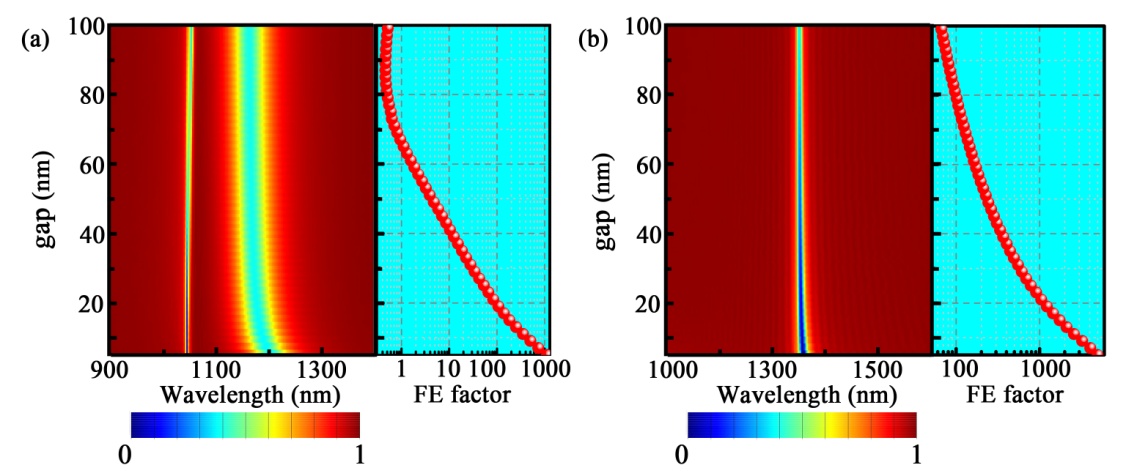


**Supplementary Figure S5 Simulated reflection spectra and FE factors of BNAs with MIM configuration as a function of the bowtie gap size.** The parameters of the BNAs with MIM configuration: *a*_⊥_=400 nm, (**a**) *t*=5 nm, *a*_||_=1,040 nm, (**b**) *t*=200 nm, *a*_||_=1,000 nm. The field intensity is measured in the middle of the bowtie gap that is 3 nm above the top-surface level of the nanoantenna.

Supplementary Fig. S6a shows the reflection spectra of the BNAs with MIM configuration and parallel coupling as a function of the superstrate refractive index. When the refractive index changes from 1.0 to 1.4, both GSP mode and LPM make a redshift. The GSP mode exhibits a wide linewidth and low wavelength sensitivity, resulting in a much lower FOM (6.3) than that based on the LPM. The sensing performance of the BNAs with the orthogonal plasmonic-photonic coupling is shown in Supplementary Figs. S6b and c. When the superstrate refractive index is increased, the extinction efficiency decreases due to the weaker coupling between the GSP and , indicating that the plasmonic sensors based on the orthogonal coupling cannot work well in a wide range of refractive index of target analytes. The wavelength sensitivity is 1,000 nm/RIU and the FOM is 108.


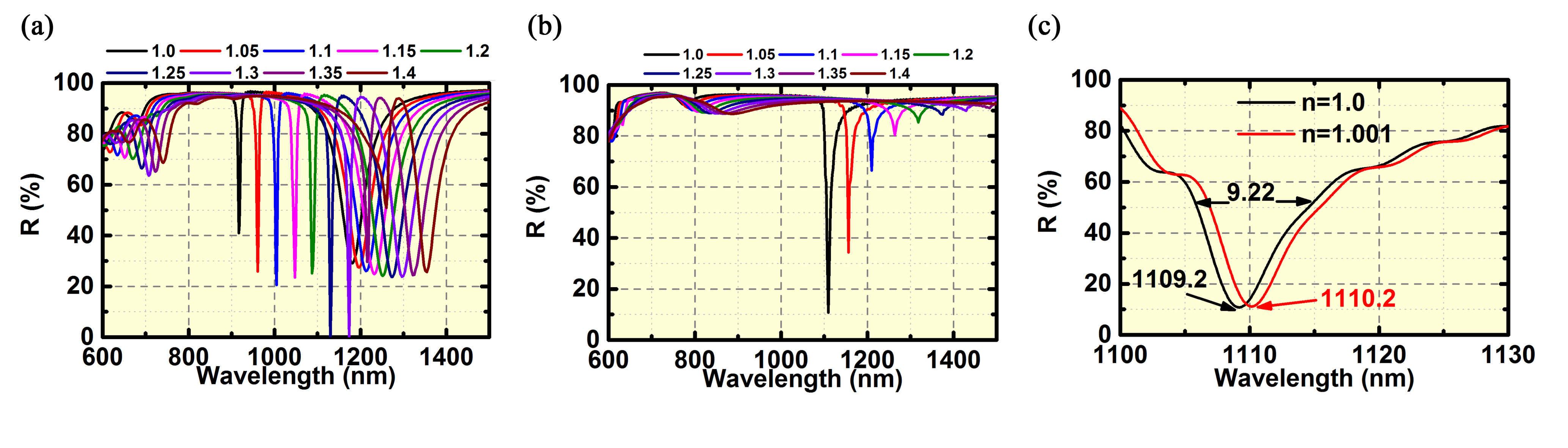


**Supplementary Figure S6.** **Refractive-index sensing of the BNAs with MIM configuration.** (a) Reflection spectra of the BNAs with MIM configuration (*t*=5 nm, *a*_⊥_=400 nm and *a*_||_=900 nm, the bowtie gap is 10 nm) as a function of the superstrate refractive index ranging from 1.0 to 1.4. Reflection spectra of the BNAs with MIM configuration (*t*=200 nm, *a*_||_=400 nm and *a*_⊥_=1,100 nm) as a function of the superstrate refractive index (**b**) changing from 1.0 to 1.4, (**c**) changing from 1.0 to 1.001. The gap size of the bowtie nanoantennas is 10 nm.

Supplementary Fig. S7 shows the amplitude and FWHM of the reflection spectra as a function of the lattice constants for the BNAs with MIM configuration. For BNAs with *t*=5 nm, a large absorption efficiency of >60% is obtained when *a*_||_ ranges from 900 to 1,100 nm (Supplementary Fig. S7a). The FWHM ranges from 3.9 to 5.8 nm. The absorption efficiency reaches >90% with the FWHM of 4.5 nm when *a*_||_=1,050 nm, leading to a Q factor of 234. However, the wavelength tunability for the BNAs with *t*=5 nm is low with the broader FWHM and lower absorption efficiency at other lattice constants. When *t*=50 nm, the high absorption efficiency with the large FWHM occurs to the BNAs with the smaller lattice constants. An increase of the lattice constants leads to the reduced linewidth, the weak plasmonic-photonic coupling efficiency, and the low absorption efficiency (Supplementary Fig. S7b). The robust orthogonal coupling in the BNAs with *t*=200 nm leads to a high-performance narrow-band absorber (Supplementary Fig. S7c). A high absorption efficiency of >80% is obtained when *a*_||_ is increased from 600 nm to 1,050 nm. However, the BNAs with orthogonal coupling have the larger FOHM and the lower Q factor than the BNAs with parallel coupling.


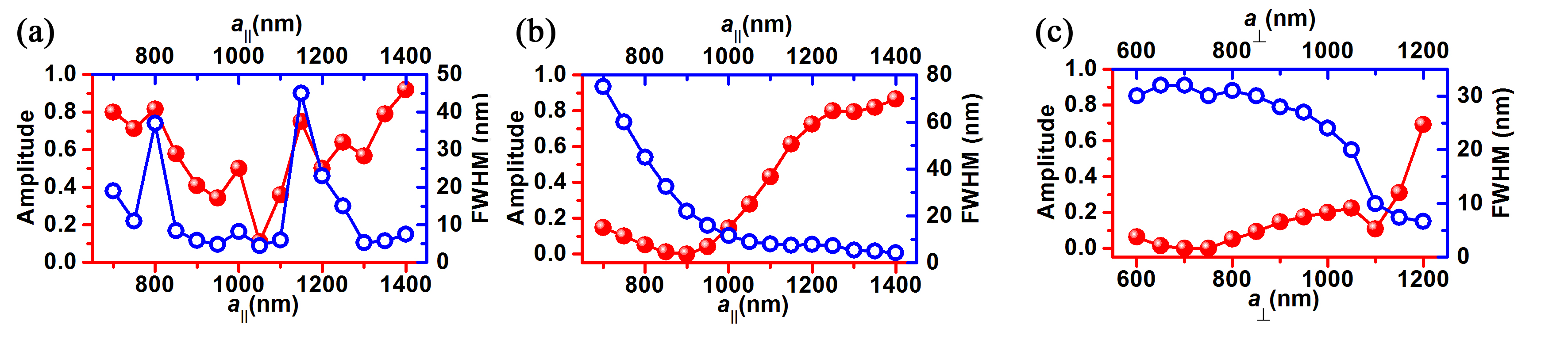


**Supplementary Figure S7 Narrow-band absorbers based on the BNAs with MIM configuration.** Amplitude and FWHM of the reflection spectra for the BNAs with MIM configuration as a function of the lattice constants with (**a**) *t*=5 nm, *a*_⊥_=400 nm and *a*_||_ ranging from 700 to 1,400 nm, (**b**) *t*=50 nm, *a*_⊥_=400 nm and *a*_||_ ranging from 700 to 1,400 nm, (**c**) *t*=200 nm, *a*_||_=400 nm and *a*_⊥_ ranging from 600 to 1,200 nm. The gap size of the bowtie nanoantennas is 10 nm.

The absorption distributions of light power in the BNAs with MIM configuration where *t*=5 nm, *a*_⊥_=400 nm and *a*_||_=1,000 nm are shown in Supplementary Fig. S8a. Due to the strong near-field coupling in the MIM structure and the intensive electric field, the highest absorption power intensity is located at the bottom of the bowtie nanoantennas. The power absorption distributions for the BNAs with MIM configuration (*t*=200 nm, *a*_||_=400 nm and *a*_⊥_=1,100 nm), where the orthogonal coupling occurs, are displayed in Supplementary Fig. S8b. The highest absorption power intensity is located around the tips of the bowtie nanoantennas. The high FE factor in the bowtie gaps leads to the much higher absorption intensity, which is consistent with the results in Fig. 6b and Figs. 8b and c.


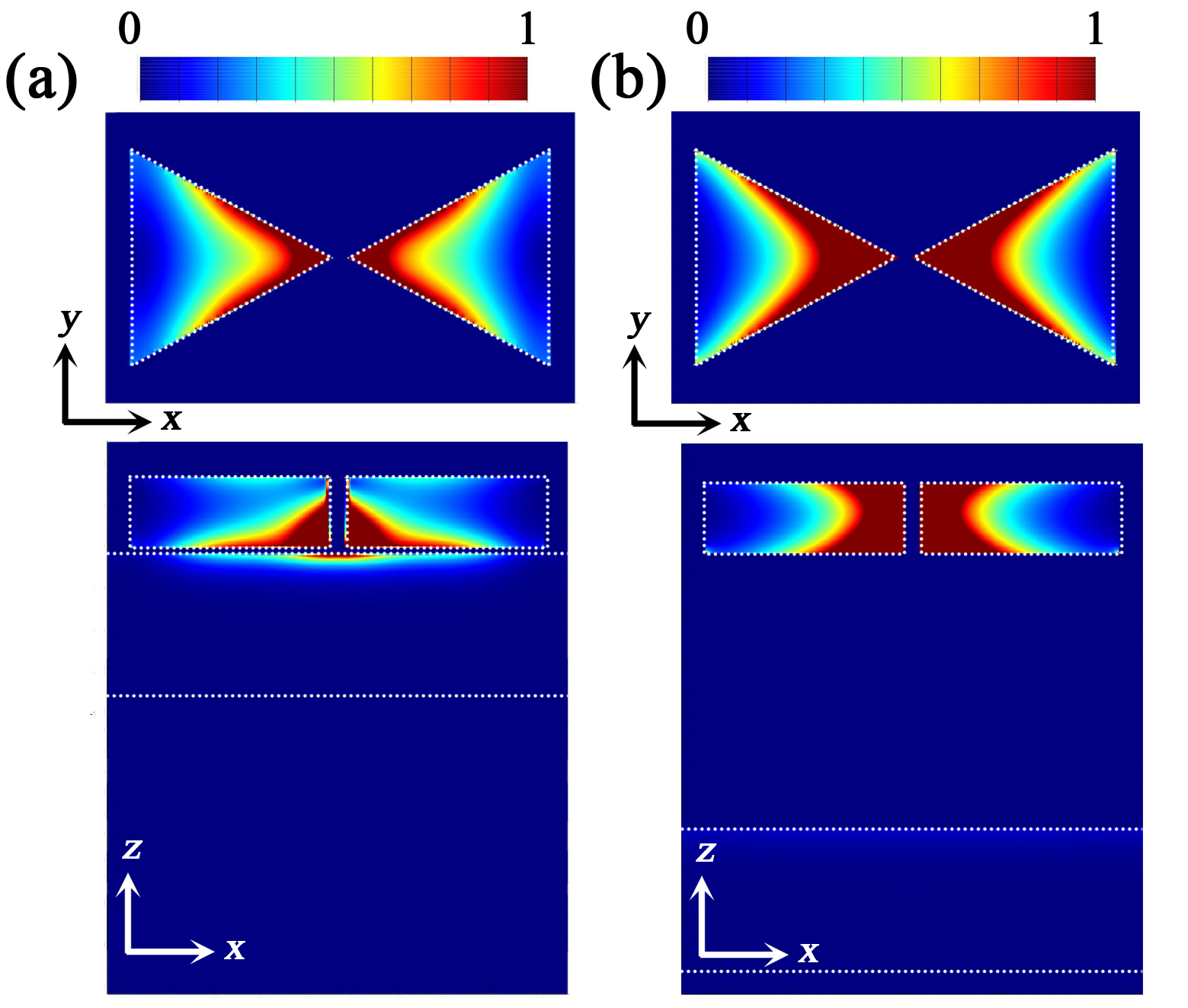


**Supplementary Figure S8 Normalized absorption power distribution maps in the BNAs with MIM configuration.** The parameters of the BNAs with MIM configuration: (**a**) *t*=5 nm, *a*_⊥_=400 nm and *a*_||_=1,000 nm at *λ*=1,007 nm, (**b**) *t*=200 nm, *a*_||_=400 nm and *a*_⊥_=1,100 nm at *λ*=1,109 nm. The distributions are in both *xy* plane (top) and *xz* plane (bottom) through the center of the bowtie nanoantennas. The maximum color scale in the bottom panel is 2 times higher than that in the top panel. The gap size of the bowtie nanoantennas is 10 nm.
